# Epithelial-to-Mesenchymal Transition and Neoangiogenesis in Laryngeal Squamous Cell Carcinoma

**DOI:** 10.3390/cancers13133339

**Published:** 2021-07-03

**Authors:** Leonardo Franz, Lorenzo Nicolè, Anna Chiara Frigo, Giancarlo Ottaviano, Piergiorgio Gaudioso, Tommaso Saccardo, Francesca Visconti, Rocco Cappellesso, Stella Blandamura, Ambrogio Fassina, Gino Marioni

**Affiliations:** 1 Department of Neuroscience DNS, Otolaryngology Section, University of Padova, 35128 Padova, Italy; leonardo.franz@aopd.veneto.it (L.F.); giancarlo.ottaviano@unipd.it (G.O.); piergiorgio.gaudioso@studenti.unipd.it (P.G.); tommaso.saccardo@studenti.unipd.it (T.S.); francesca.visconti@studenti.unipd.it (F.V.); 2 Ulss3 Serenissima, Unit of Surgical Pathology & Cytopathology, Ospedale dell’Angelo, 30174 Mestre, Italy; lorenzo.nicole@phd.unipd.it; 3 Unit of Pathology & Cytopathology, Department of Medicine (DIMED), University of Padova, 35121 Padova, Italy; ambrogio.fassina@unipd.it; 4 Department of Cardiac-Thoracic-Vascular Sciences and Public Health, Padova University, 35128 Padova, Italy; annachiara.frigo@unipd.it; 5 Department of Medicine (DIMED), Section of Pathological Anatomy, University of Padova, 35121 Padova, Italy; rocco.cappellesso@aopd.veneto.it (R.C.); stella.blandamura@unipd.it (S.B.)

**Keywords:** laryngeal squamous cell carcinoma, epithelial–mesenchymal transition, N-cadherin, Slug, angiogenesis, CD105, prognosis

## Abstract

**Simple summary:**

The mechanism of epithelial–mesenchymal transition is fundamental for carcinogenesis, tumor progression, cancer cell invasion, metastasis, recurrence, and therapy resistance, resulting in cellular junction degradation and increased cellular motility. The same factors that drive epithelial cells toward a mesenchymal phenotype may also drive endothelial cells toward a proangiogenic phenotype. This study aimed to investigate a potential interplay between epithelial–mesenchymal transition and angiogenesis in laryngeal carcinoma. In our study, univariate Cox regression identified pN+ status and Slug expression as predictive of disease-free survival, while a trend toward significance emerged for CD105-assessed microvessel density and N-cadherin expression. In the multivariate Cox regression model, pN-status, Slug, and N-cadherin expressions retained their significant values in predicting disease-free survival. Data from our study support the hypothesis of a mutual concurrence of epithelial–mesenchymal transition and angiogenesis in the development of an aggressive phenotype in laryngeal squamous cell carcinoma.

**Abstract:**

The mechanism of epithelial–mesenchymal transition (EMT) is fundamental for carcinogenesis, tumor progression, cancer cell invasion, metastasis, recurrence, and therapy resistance, comprising important events, such as cellular junction degradation, downregulation of epithelial phenotype markers, overexpression of mesenchymal markers, and increase in cellular motility. The same factors that drive epithelial cells toward a mesenchymal phenotype may also drive endothelial cells toward a proangiogenic phenotype. The aim of this exploratory study was to investigate a potential interplay between EMT and angiogenesis (quantified through CD105 expression) in laryngeal carcinoma (LSCC). CD105-assessed microvessel density (MVD) and EMT markers (E-cadherin, N-cadherin, Snail, Slug, Zeb1, and Zeb2) were assessed on 37 consecutive LSCC cases. The univariate Cox regression model identified pN+ status (*p* = 0.0343) and Slug expression (*p* = 0.0268) as predictive of disease-free survival (DFS). A trend toward significance emerged for CD105-assessed MVD (*p* = 0.0869) and N-cadherin expression (*p* = 0.0911). In the multivariate Cox model, pN-status, Slug, and N-cadherin expressions retained their significant values in predicting DFS (*p* = 0.0346, *p* = 0.0430, and *p* = 0.0214, respectively). Our data support the hypothesis of a mutual concurrence of EMT and angiogenesis in driving LSCC cells toward an aggressive phenotype. To better characterize the predictive performance of prognostic models based on EMT and angiogenesis, further large-scale prospective studies are required.

## 1. Introduction

The mechanism of epithelial–mesenchymal transition (EMT) is fundamental for carcinogenesis, tumor progression, cancer cell invasion, metastasis, recurrence, and therapy resistance, comprising important events such as the degradation of cellular junctions, morphological changes in the neoplastic cell, downregulation of epithelial phenotype markers, overexpression of mesenchymal markers, and an increase in cellular motility and matrix metalloproteinases [1,2,3,4,5]. These events involve molecular reprograming of the cell centered on a decrease in the level of E-cadherin [6], a key component of the adherens junction complexes, and an increase in the level of N-cadherin, involved in pathways enhancing cell survival and migration. This process is called cadherin switching [7]. The E-cadherin downregulation is carried out by the nuclear factors Snail, Twist, Zeb1, and Zeb2, which directly bind to its gene promoter [8,9].

The same factors that drive epithelial cells toward a mesenchymal phenotype may also drive endothelial cells toward a proangiogenic phenotype [1]. Angiogenesis is a process fundamental to tumor growth. Tumor angiogenesis is currently considered the result of an imbalance between pro- and anti-angiogenic factors produced by both malignancy and normal cells [10]. The assessment of microvessel density (MVD) is a well-established approach to obtaining quantitative data about neoplastic angiogenesis processes, and it is currently often based on the immune staining of vascular endothelial cell markers [11]. Endoglin (CD105) is a proliferation-associated and hypoxia-inducible protein abundantly expressed in angiogenic endothelial cells [12]. CD105 has also very recently been confirmed as having a promising role in evaluating and quantifying neoangiogenesis in laryngeal squamous cell carcinoma (LSCC) [13].

The identification of possible tumor biomarkers exhibiting high sensitivity and specificity could contribute to an early diagnosis and monitoring of the therapeutic response, especially in advanced LSCC patients.

The main aim of this exploratory study was to investigate the existence of a potential interplay between EMT (by assessing the immunohistochemical expression of E-cadherin, N-cadherin, Snail, Slug, Zeb1, and Zeb2) and angiogenesis (quantified through the image analysis of immunohistochemical expression of CD105) in LSCC. A secondary aim was to verify whether considering a combination of EMT and angiogenesis variables might improve prognostic accuracy compared to the traditional clinical–pathological prognostic tools.

## 2. Materials and Methods

### 2.1. Patients

This study was conducted in accordance with the principles of the Helsinki Declaration. Data were examined in compliance with Italian privacy and sensitive data laws and the in-house rules of Padova University’s Otolaryngology Section. All patients preoperatively signed a consent form for disclosure of privacy in managing personal data for scientific purposes. In particular, they consented “to the use of their clinical data for scientific research purposes in the medical, biomedical and epidemiological fields, also in order to be recalled in the future for follow-up needs”. Before undergoing surgery, all patients included in the study signed a detailed informed consent form.

The investigation involved 37 consecutive cases (33 males and 4 females; mean age 63.6 ± 8.7 years) of LSCC treated with primary surgery. As in the recommendations adopted for LSCC at our institution, all patients had undergone microlaryngoscopy with laryngeal biopsy, upper aerodigestive tract endoscopy, neck ultrasonography (with or without fine needle aspiration cytology), head and neck contrast-enhanced computerized tomography (CT), and/or magnetic resonance imaging, chest X-ray, and liver ultrasonography.

All patients underwent laryngeal surgery with unilateral or bilateral cervical lymph node dissection at the Otolaryngology Section of Padova University. Pathological findings warranted postoperative adjuvant RT (with or without concomitant chemotherapy) in 15 cases in accordance with current guidelines (NCCN, 2020). Table 1 provides details of patients’ clinical and pathological features, based also on the 8th edition of the TNM Classification of malignant tumors [14]. No distant metastases (M) were detected at diagnosis. As previously reported [9], at our institution, the clinical follow-up after treatment (adjustable to patients’ individual characteristics) was scheduled as follows: (i) once a month for the 1st year; (ii) every 2 months in the 2nd year; (iii) every 3 months in the 3rd year; (iv) every 4 months in the 4th year; (v) every 6 months in the 5th year; and (vi) every 12 months thereafter. Contrast-enhanced CT of the neck, total-body positron emission tomography, chest CT, and neck and liver ultrasonography were repeated if clinically indicated. The median follow-up was 24 months (interquartile range (IQR) = 13–63 months).

### 2.2. Immunohistochemistry

#### 2.2.1. EMT Markers

**Figure 1:**
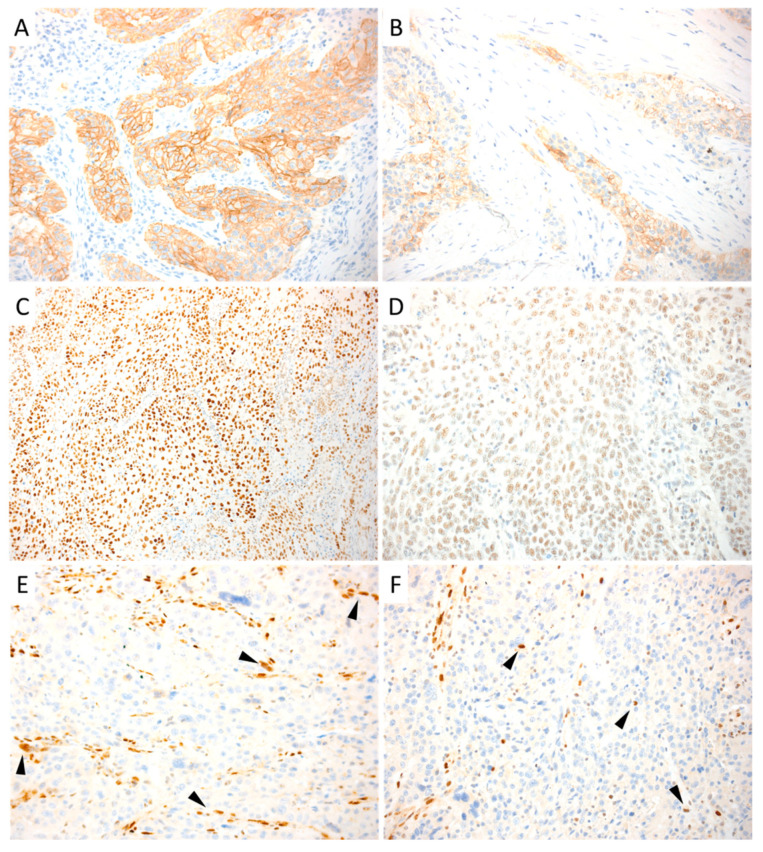
Representative cases of positive immunohistochemistry stain. Cancer cells showing strong membrane immunoreaction for E-cadherin (**A**) and N-cadherin (**B**); nuclear expression for Snail (**C**), Slug (**D**), Zeb1 (**E**), and Zeb2 (**F**). Note that Zeb1 and Zeb2 resulted in a patchy stain pattern in all cases (arrowheads). A, B, C, D, E, F: Original magnification: 100×.

Immunohistochemical staining was performed using the Bond Polymer Refine Detection kit (Leica Biosystems, Newcastle upon Tyne, UK) in the fully automated BondmaX system (Leica Biosystems) on 4-µm-thick formalin fixed, paraffin-embedded sections cut from each tumor sample. Sections were pretreated using heat-mediated antigen retrieval with sodium citrate buffer (pH 6, epitope retrieval solution 1; Leica Biosystems) for 30 min at 99 °C. They were then incubated with the primary antibodies for E-cadherin (clone NCH38, 1:200 dilution; Dako Cytomation, Glostrup, Denmark), N-cadherin (clone 6G11, 1:100 dilution; Dako Cytomation), Snail (Polyclonal, 1:100 dilution; Novus Biologicals, Littleton, CO), Zeb1 (Polyclonal, 1:100 dilution; Santa Cruz Biotechnology), and Zeb2 (Polyclonal, 1:100 dilution; Santa Cruz Biotechnology). The slides were ultimately slightly counterstained with hematoxylin. Appropriate positive and negative controls were used. Only membrane immunoreactions were considered to assess E-cadherin and N-cadherin expression, whereas for Snail, Zeb1, and Zeb2, only the nuclear stain was considered. The percentage of immunostained neoplastic cells was assessed. Representative images of each staining are shown in Figure 1.

#### 2.2.2. CD105-Assessed Microvessel Density (MVD)

**Figure 2:**
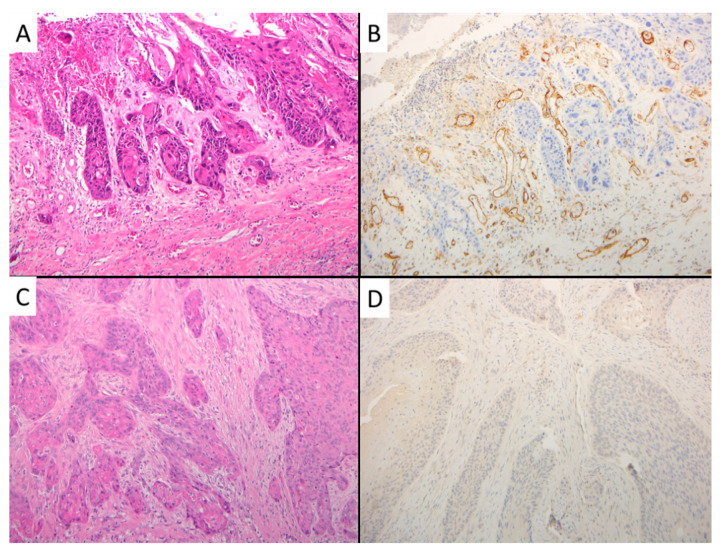
Representative cases with matched microvessel stain with CD105. (**A**) Squamous cell carcinoma with moderate differentiation and high MVD as shown by CD105 stain (**B**). (**C**) Squamous cell carcinoma with moderate differentiation and low MVD as shown by CD105 stain (**D**). A, C: Hematoxylin and eosin stain. A, B, C, D: Original magnification: 100×.

The antibody used was CD105 (monoclonal mouse antibody, cloneSN6h, diluted 1:10; Dako, Glostrup, Denmark) (Figure 2). Appropriate positive controls were run concurrently, according to the manufacturer’s protocols. The primary antibody was replaced by phosphate buffer saline as a negative control. All images were analyzed on a workstation consisting of a conventional Zeiss Axioskop light microscope (Zeiss, Jena, Germany) with a Peltier-cooled color digital video camera (MicroPublisher 5.0 RTV, QImaging, Surrey, Canada) connected to a personal computer with the Image-Pro Plus 7 for Windows IA program (Media Cybernetics, Bethesda, MD). In all cases, 1378 × 954-μm areas of tumor tissue were comprehensively examined using a 495-point sampling grid superimposed by the program on the image acquired with a ×50 field of view. The percentage of fields occupied by CD105-assessed microvessels (area fraction) was calculated for each specimen.

### 2.3. Statistical Analysis

The data have been presented as means and standard deviations, medians and IQRs for quantitative variables, and as counts and percentages for the categorical variables. The recurrence-free survival of LSCC according to conventional clinical–pathological features, CD105-assessed MVD, and EMT variables was analyzed with univariate Cox regression models. The results of the Cox regression are expressed as *p*-values, hazard ratios, and 95% confidence intervals (CI). The time to LSCC recurrence was calculated as the time from completing treatment for the LSCC to disease recurrence, or to last follow-up for patients experiencing no relapse. The predictors found to be statistically significant at the 0.10 level in the univariate Cox regression analyses were input into a multivariate regression analysis. The proportionality assumption of the Cox models was checked with a Kolmogorov-type supremum test using 1000 resamplings. The predictive ability of the multivariate survival model was evaluated with Uno’s concordance statistic [15] estimated at selected timepoints: 6, 12, 18, and 24 months. Uno’s concordance standard error was estimated with 100 perturbation resamplings, and the 95% CI was calculated considering the normal approximation. We also estimated the area beneath the curves (AUCs) of the time-dependent receiver operating characteristic (ROC) curves with the inverse probability of censoring weighting (IPCW) [15] along with the 95% pointwise confidence limits calculated using 50 perturbation resamplings. The association of EMT marker expressions and CD105-assessed MVD with conventional clinical-pathological variables as pT- and pN-status was assessed using the Mann–Whitney *U* test. Spearman’s rank correlation with 95% confidence interval calculated with the Fisher *z*-transformation was applied for the correlation between EMT markers and CD105-assessed MVD. Unless otherwise indicated, a *p*-value of <0.05 was considered indicative of statistical significance. The statistical analyses were performed with SAS 9.4 for Windows (SAS Institute Inc., Cary, NC, USA).

## 3. Results

### 3.1. Clinical Outcomes

Twenty-one out of 37 LSCC patients (56.8%) experienced no disease recurrence after a median follow-up period of 61 months (IQR = 35–109 months), while 16 (34.1%) showed a disease relapse after a median follow-up period of 11.5 months (IQR = 6–17.5 months).

### 3.2. Univariate Survival Analysis

Cigarette smoking and alcohol abuse have been investigated as potential predictors for the time to LSCC recurrence, but we did not find a statistically significant association (rate of recurrence in previous or actual smokers vs. in non-smokers *p* = 0.8138, in alcohol abusers vs. in non-abusers *p* = 0.6099). Table 1 shows the association between CD105-assessed MVD, EMT proteins, conventional pathological variables, and DFS. The univariate Cox regression model identified pN+ status (HR 3.418, 95% CI 1.095–10.668; *p* = 0.0343), and Slug expression (HR 0.946, 95% CI 0.901-0.994; *p* = 0.0268) as predictive of DFS. A trend toward significance in predicting DFS emerged for CD105-assessed MVD (HR 1.036, 95% CI 0.995–1.079; *p* = 0.0869), and N-cadherin expression (HR 1.018, 95% CI 0.997–1.040; *p* = 0.0911).

### 3.3. Multivariate Prognostic Model

Variables that showed a *p*-value of <0.10 in the univariate analysis were included in the multivariate model. As a result, pN-status, CD105-assessed MVD, N-cadherin, and Slug expressions were considered (Table 2).

In the multivariate Cox regression model, pN-status (pN+ vs. pN0), N-cadherin, and Slug expressions retained their significant values in predicting DFS (HR 3.717, 95% CI 1.100–12.55; *p* = 0.0346, HR 1.027, 95% CI 1.101–1.054; *p* = 0.0430, and HR 0.924, 95% CI 0.865–0.988; *p* = 0.0214, respectively).

**Figure 3:**
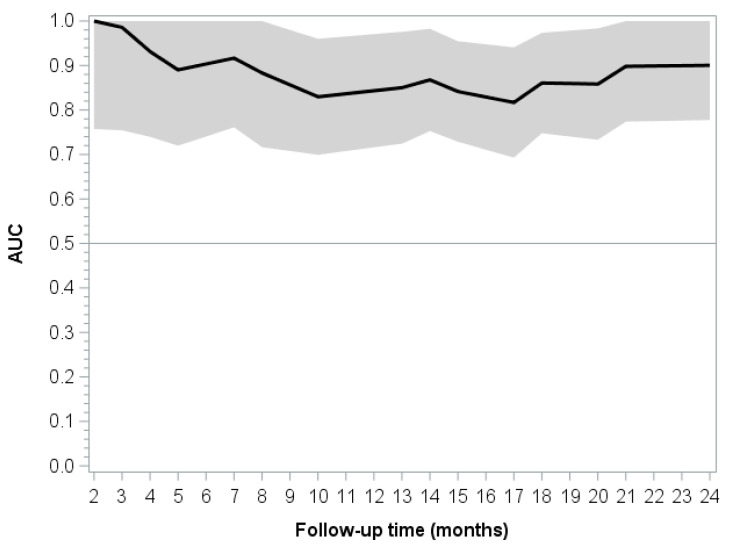
Time-dependent AUCs and 95% confidence intervals.

The integrated time-dependent area beneath the curve for the multivariate model was 0.8901 (see also Figure 3). Uno’s concordance statistics calculated for the multivariate model at different time points are summarized in Table 3. These results show that the predictive ability decreases as time passes, reaching a steady state at the end.

### 3.4. Association between EMT Proteins, Angiogenesis, and Other Clinical-Pathological Variables in LSCC

Mann–Whitney *U* test ruled out significant associations of considered EMT markers expressions and CD105-assessed MVD with conventional clinical–pathological variables such as pT-status (pT1-2 vs. pT2-3) and pN-status (pN+ vs. pN0) (*p* > 0.05).

A significant positive correlation was found between Slug and E-cadherin (Spearman’s rank correlation; rho = 0.39, 95% CI: 0.08–0.63, *p* = 0.0162) and between Zeb2 and Snail (Spearman’s rank correlation; rho = 0.44, 95%CI: 0.13–0.67, *p* = 0.0062). A trend toward a significant correlation was found between two EMT markers (Snail and Zeb2) and CD105-assessed MVD (Spearman’s rank correlation, rho = 0.30, 95% CI: −0.02–0.57, *p* = 0.07 and rho = 0.27, 95% CI: −0.06–0.54, *p* = 0.11, respectively). 

## 4. Discussion

The EMT program has also recently been investigated in LSCC [9]. Greco et al. [16] evaluated the clinical significance of E-cadherin, N-cadherin, β-catenin, α-catenin, γ-catenin, caveolin-1, and vimentin in a cohort of 82 patients with LSCC, treated with surgery, with or without adjuvant therapy. Cytoplasmic β-catenin overexpression was independently associated with a longer disease-specific survival, and E-cadherin overexpression was found to be an independent risk factor for poor overall survival (OS). Zuo et al. [17] considered 57 surgical samples from patients with LSCC to investigate the biological relationship between EMT, hypoxia and glycolitic shift, and their prognostic impact. Hypoxia was supposed to enhance cell invasion and migration, inducing a glycolitic shift and an EMT phenotype, characterized by increased glucose metabolism such as glucose transporter 1 (GLUT-1), N-cadherin and vimentin expression, and decreased E-cadherin. Patients’ survival rates with the positive expression of GLUT-1, vimentin, and N-cadherin were significantly poorer compared with those with negative expression of GLUT-1, vimentin, and N-cadherin, leading to the conclusion that hypoxia might promote laryngeal cancer cell invasion and migration via EMT. Zhu et al. [18] studied immunohistochemistry, E-cadherin, N-cadherin, β-catenin, and Zeb2 protein expression in a cohort of 76 patients with operable LSCC. They found that negative E-cadherin expression and positive N-cadherin, β-catenin, and Zeb2 expression were associated with a reduced OS. Very recently, Zhang et al. [19] analyzed in 127 LSCC tissue samples the relationship between sex-determining region Y-related high-mobility-group box 4 (SOX4) and EMT, and their relationship with clinical-pathological factors and related prognosis. The positive expression rate of SOX4 and Slug in LSCC was related to pathological differentiation, lymphatic invasion, and pathological tumor node metastasis. The expression rates of Zeb1, Twist, E-cadherin, N-cadherin, and β-catenin in LSCC correlated with lymphatic invasion and pathological tumor node metastasis.

The concept that EMT and angiogenesis may mutually contribute to the development of an aggressive tumor phenotype seems to be supported by both biological and clinical data. It has been reported that EMT is necessary for angiogenesis itself to be completed. Initiation of vascular sprouting requires the endothelial tip cells to acquire a mesenchymal phenotype, losing apical-basal polarity and thus becoming able to degrade basement membrane and extracellular matrix, and to migrate [1]. In a cancer microenvironment, the increased growth rate of tumor cells leads to the development of hypoxic areas that are relatively separated from the nearest vessels. Tumor cells respond to such stress by overexpressing angiogenesis factors such as vascular endothelial growth factor (VEGF) and acquiring a migration-oriented phenotype, which is the result of an EMT process [20]. From a clinical viewpoint, it has been found that, in several malignancies, a key determinant of disease progression was vascular invasion, which was eased by both neoangiogenesis and the acquisition of a mesenchymal phenotype [21]. Moreover, EMT itself enables cells to migrate through the peritumoral stroma, resulting in the possibility of infiltrating beyond the tumor boundaries. Cancer cells undergoing EMT may even directly form part of the desmoplastic stroma as in non-small cell lung cancer [22].

To the best of our knowledge, this study is the first to investigate the relationship between EMT and CD105-assessed MVD in LSCC. The main strength of our investigation lies in the homogeneity of the patient population since: (i) all patients underwent surgery; (ii) their surgical treatment was performed by the same team; (iii) only squamous cell carcinomas located in the larynx were considered; and (iv) clinical-radiological follow-up criteria were defined. In addition, a computer-based IA system was used to ensure a highly accurate, precise, and reproducible analysis of the immunostained slides in terms of CD105-assessed MVD. Over the course of about 15 years, the pathologists in our research group have gained plenty of experience in assessing MVD from the immunohistochemical expression of CD105 in head and neck malignancies using various IA techniques, and also in association with oncogenes or tumor suppressors [9,13]. The main weaknesses of our study concern the retrospective setting and limited number of cases considered.

As previously widely reported, in our series, patients with a pN+ lymph node status had a lower DFS. Furthermore, the prognostic role of Slug and N-cadherin expression, as long as the marginal confirmation of CD105-assessed MVD was a negative predictive factor, seemed to support the hypothesis that both angiogenesis and EMT may act as determinants of an aggressive phenotype in LSCC. The multivariate analysis, besides confirming pN+ status and Slug overexpression as significant independent prognostic factors in regard to DFS, seemed to depict the simultaneous consideration of both EMT and angiogenesis markers as a potentially promising strategy to stratify clinical recurrence risk in patients with LSCC. On the other hand, this study only found a trend toward a positive correlation between CD105-assessed MVD and both Snail and Zeb2 expressions, probably due to the limited sample size of our LSCC series. These marginal significances may be explained by the biological interactions of the EMT cascade: both Snail and Zeb2 are well-ascertained effectors of the EMT process in many cancer types, acting as nuclear factors downstream of the Wnt/ß-catenin pathway [23,24,25]. Such a pathway also allows actin cytoskeleton to be remodeled during the biological cascade in response to hypoxia [26]. As is well known, hypoxia also stimulates angiogenesis via the overexpression of proangiogenic factors such as the VEGF receptor family [23], therefore resulting in an increased expression of a marker of activated endothelium, such as CD105. On the other hand, Zeb2 has pleotropic biological functions beyond the EMT process, also directly upregulating the expression of genes involved in angiogenesis, such as VEGF, thus resulting in endothelial cell activation and proliferation [25].

## 5. Conclusions

In conclusion, our data support the hypothesis of a mutual concurrence of EMT and angiogenesis in the development of an aggressive phenotype in LSCC. To better characterize the predictive performance of prognostic models based on EMT and angiogenesis markers in stratifying LSCC recurrence risk, further large-scale prospective studies are required.

## Figures and Tables

**Table 1:** Descriptive statistics of CD105-assessed MVD, EMT variables (E-cadherin, N-cadherin, Snail, Slug, Zeb1, and Zeb2 expressions), and conventional clinical-pathological features according to laryngeal squamous cell carcinoma (LSCC) recurrence, and univariate Cox regression analysis results for LSCC recurrence.

LSCC Recurrence					
Variable	No (No. = 21)	Yes (No. = 16)	As a Whole (No. = 37)	HR (95% CI)	*p*-Value
Gender					
Male	19 (90.5)	14 (87.5)	33 (89.2)	1	0.6032
Female	02 (9.5)	02 (12.5)	04 (10.8)	1.48 (0.336–6.542)	
Age (years)					
Mean (SD)	61.52 (9.39)	66.31 (6.99)	63.59 (8.67)	1.040 (0.982–1.103)	0.1825
Median (IQR)	61.00 (58.00–68.00)	66.00 (61.50–71.50)	64.00 (59.00–70.00)		
pT					
pT1-T2	05 (23.8)	04 (25.0)	09 (24.3)	1	0.9012
pT3-T4	16 (76.2)	12 (75.0)	28 (75.7)	0.931 (0.300–2.889)	
pN-status					
pN0	12 (57.1)	04 (25.0)	16 (43.2)	1	0.0343
pN+	09 (42.9)	12 (75.0)	21 (56.8)	3.418 (1.095–10.668)	
CD105-assessed MVD %					
Mean (SD)	5.01 (6.13)	11.15 (9.99)	7.66 (8.48)	1.036 (0.995–1.079)	0.0869
Median (IQR)	3.32 (0.01–6.67)	9.87 (5.43–13.82)	5.70 (2.12–10.43)		
E-Cadherin expression %					
Mean (SD)	43.10 (36.52)	29.69 (31.44)	37.30 (34.61)	0.992 (0.976–1.007)	0.3029
Median (IQR)	40.00 (10.00–80.00)	15.00 (0.00–60.00)	20.00 (5.00–70.00)		
N-Cadherin expression %					
Mean (SD)	3.81 (13.22)	13.13 (21.12)	7.84 (17.46)	1.018 (0.997–1.040)	0.0911
Median (IQR)	0.00 (0.00–0.00)	0.00 (0.00–25.00)	0.00 (0.00–5.00)		
Snail expression %					
Mean (SD)	11.67 (14.08)	23.13 (28.45)	16.62 (21.92)	1.015 (0.994–1.036)	0.1551
Median (IQR)	10.00 (0.00–20.00)	5.00 (0.00–55.00)	5.00 (0.00–30.00)		
Slug expression %					
Mean (SD)	30.48 (31.38)	5.00 (9.13)	19.46 (27.30)	0.946 (0.901–0.994)	0.0268
Median (IQR)	20.00 (0.00–50.00)	0.00 (0.00–7.50)	10.00 (0.00–30.00)		
Zeb1 expression %					
Mean (SD)	1.90 (4.87)	3.75 (5.63)	2.70 (5.22)	1.028 (0.952–1.109 )	0.4798
Median (IQR)	0.00 (0.00–0.00)	0.00 (0.00–5.00)	0.00 (0.00–5.00)		
Zeb2 expression %					
Mean (SD)	3.57 (6.73)	8.44 (9.78)	5.68 (8.43)	1.042 (0.991–1.096)	0.1073
Median (IQR)	0.00 (0.00–5.00)	5.00 (0.00–17.50)	0.00 (0.00–10.00)		

HR: hazard ratio; CI: confidence interval.

**Table 2:** Multivariate Cox regression analysis results on the variables which showed a *p*-value of <0.10 in the univariate analysis.

Variable	HR (95% CI)	*p*-Value
pN status	3.717 (1.100–12.558)	0.0346
CD105-assessed MVD %	1.013 (0.973–1.054)	0.5291
N-Cadherin %	1.027 (1.001–1.054)	0.0430
Slug %	0.924 (0.865–0.988)	0.0214

HR: hazard ratio; CI: confidence interval.

**Table 3:** Uno’s concordance coefficient for the multivariate model at different timepoints.

Time (Months)	Uno’s Concordance Coefficient (SE)	95% CI
6	0.8949 (0.0797)	0.7387–1.0000
12	0.8320 (0.0951)	0.6456–1.0000
18	0.8120 (0.0884)	0.6387–0.9853
24	0.8252 (0.0800)	0.6684–0.9820

SE: standard error; CI: confidence interval.
